# Sensitivity analysis of a mathematical model of Alzheimer's disease progression unveils important causal pathways

**DOI:** 10.3389/fninf.2025.1590968

**Published:** 2025-07-23

**Authors:** Seyedadel Moravveji, Halima Sadia, Nicolas Doyon, Simon Duchesne

**Affiliations:** ^1^Department of Mathematics and Statistics, Université Laval, Quebec, QC, Canada; ^2^Medics Laboratory, Quebec Heart and Lung Institute, Quebec, QC, Canada; ^3^Faculty of Medecine, Université Laval, Quebec, QC, Canada; ^4^CERVO Brain Research Centre (Centre de recherche CERVO sur le cerveau, le comportement et la neuropsychiatrie), Quebec, QC, Canada; ^5^Department of Radiology and Nuclear Medicine, Université Laval, Quebec, QC, Canada

**Keywords:** Alzheimer's disease, mathematical models, neural density, sensitivity analysis, amyloid beta, tau proteins, APOE, synergy

## Abstract

**Introduction:**

Mathematical models serve as essential tools to investigate brain aging, the onset of Alzheimer's disease (AD) and its progression. By studying the representation of the complex dynamics of brain aging processes, such as amyloid beta (Aβ) deposition, tau tangles, neuro-inflammation, and neuronal death. Sensitivity analyses provide a powerful framework for identifying the underlying mechanisms that drive disease progression. In this study, we present the first local sensitivity analysis of a recent and comprehensive multiscale ODE-based model of Alzheimer's Disease (AD) that originates from our group. As such, it is one of the most complex model that captures the multifactorial nature of AD, incorporating neuronal, pathological, and inflammatory processes at the nano, micro and macro scales. This detailed framework enables realistic simulation of disease progression and identification of key biological parameters that influence system behavior. Our analysis identifies the key drivers of disease progression across patient profiles, providing insight into targeted therapeutic strategies.

**Methods:**

We investigated a recent ODE-based model composed of 19 variables and 75 parameters, developed by our group, to study Alzheimer's disease dynamics. We performed single- and paired-parameter sensitivity analyses, focusing on three key outcomes: neural density, amyloid beta plaques, and tau proteins.

**Results:**

Our findings suggest that the parameters related to glucose and insulin regulation could play an important role in neurodegeneration and cognitive decline. Second, the parameters that have the most important impact on cognitive decline are not completely the same depending on sex and APOE status.

**Discussion:**

These results underscore the importance of incorporating a multifactorial approach tailored to demographic characteristics when considering strategies for AD treatment. This approach is essential to identify the factors that contribute significantly to neural loss and AD progression.

## 1 Introduction

Alzheimer's disease (AD) is a progressive neurodegenerative disorder characterized by the accumulation of amyloid beta (*Aβ*) plaques and tau neurofibrils, leading to neuronal cell death and loss of cognitive function. Recently approved *Aβ* antibody treatments, while successful in removing *Aβ*, are not sufficient to stop the progression of neurodegeneration. Hence, treatment for AD is likely to require a more comprehensive approach combining interventions that address not only *Aβ* and tau but also other factors that contribute to decline, such as cardiovascular and metabolic health.

To be effective, such a multifaceted strategy must rest on a better understanding of the complex etiology and the roles of each factor in its trajectory, along with a mechanism by which interventions can be tailored to individual health profiles. This requires a larger, more integrated perspective that encompasses all of the various abnormalities that are present in AD, such as aberrant production and accumulation of amyloid and tau proteins; inflammation surge; neuronal dysfunction and death; and, inevitably, cognitive and behavioral impairment. Although these abnormalities are extensively researched individually, few large-scale integrative efforts have been made. Experimentally, assessing a global theory is logistically impossible because of the substantial number of variables involved. In addition, the design of multifactorial trials in preclinical models or clinical cohorts is faced with ethical, practical, and financial problems due to the long duration of neurodegeneration. In particular, we acknowledge the role of high-throughput experimental platforms, longitudinal cohort studies, and machine learning in (AD) research, which have enabled the discovery of diagnostic biomarkers and disease-stage classifiers. For instance, recent studies have successfully applied machine learning techniques to classify stages of AD using neuro-imaging data, highlighting their utility for stratification and detection tasks (Khan et al., 2022, 2023). However, while such data-driven techniques are invaluable for detection and stratification, they are often limited in their ability to translate in human studies (high-throughput experimental platforms), capture the numerous changes occurring in both aging and pathological aging (longitudinal cohort studies) or understand the various causal mechanisms of disease progression (machine learning). In contrast, our mechanistic ODE-based model allows hypothesis-driven exploration of biological interactions, allowing the testing of causal pathways through sensitivity analyses.

Computational models, on the other hand, are computer programs that represent a complex nonlinear system, that are based on domain knowledge but that are trained and validated using real or simulated data. They can combine the description of several entities of interest into a complete theoretical framework, allowing for the generation and testing of numerous numerically verifiable hypotheses. Therefore, mechanistic computer models of the aging brain can handle many factors at different levels of abstraction, circumventing many of the limitations of preclinical and clinical trials, while offering an *in-silico* environment for testing interventions and proposing personalized therapeutic regimens.

**Figure 1:**
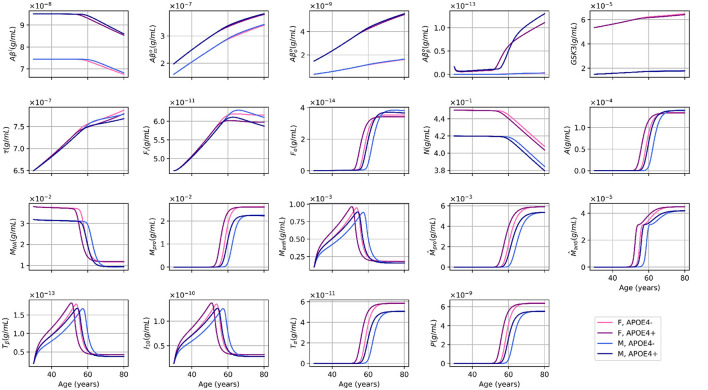
Age-dependent evolution of variable concentrations for different sex and APOE4 status combinations, as simulated by our standard model. This figure is adapted from Chamberland et al. (2024), with permission.

In a previous paper (Chamberland et al., 2024), we created such a model at two levels of abstraction, the nanoscale (proteins, genes) and microscale (cells, tissues) Figure 1. We used ordinary differential equations (ODE) to describe the connections between different cell types (e.g., neurons, astrocytes, macrophages, and microglia) and concentrations of proteins and protein aggregates that were pathological in nature (such as *Aβ* monomers, oligomers, plaques; tau filaments and tangles), or related to inflammatory processes (such as anti-inflammatory cytokines and chemokines).

Missing from this previous study was a robust analysis of model sensitivity. In the context of ODE models, sensitivity analysis can identify the most influential parameters related to specific outcomes of interest, such as *Aβ* and tau concentrations or neuronal loss. It can also help investigate how simultaneous changes in several parameters interact and may impact outcomes of interest in a non-linear manner. Furthermore, sensitivity analysis may reveal whether a model is well-constructed or not, as parameters with no impact on outcomes can be considered superfluous.

In our earlier scoping review of mathematical models of AD (Moravveji et al., 2024), we discussed the importance of conducting such sensitivity analyses in general, and in particular, to uncover key processes related to AD. Among the examples from the literature, we mentioned a study by Proctor et al. (2013) that used sensitivity analysis to investigate the impact of parameters associated with *Aβ* oligomers formation and tau aggregation on neuronal density dynamics. The researchers found strong links between these parameters and the neuronal density data. However, the analysis performed by Kyrtsos and Baras (2015) in their system biology model highlighted the importance of parameters related to neuro inflammation, oxidative stress, and synaptic dysfunction in shaping the path of neuronal density. Similarly, the findings of the sensitivity analysis by Bertsch et al. (2020) provided valuable information on key biological processes that drive the pathogenesis of AD, such as *Aβ* and tau production, clearance, and propagation. The authors also used the results of the sensitivity analysis to fine-tune their model and explore different scenarios of disease progression, accounting for the variability in parameter values and their impact on model output.

In this work, we propose a quantitative sensitivity analysis of the (Chamberland et al., 2024) model. We examined how changes in parameters affect important outcomes such as neuronal count (*N*) and *Aβ* / τ protein concentrations over adult lifespan (30 to 80 years). We first performed single-parameter and then paired-parameter variations and determined their influence on the outcomes.

## 2 Methods

### 2.1 Overview

To better understand how model parameters influence biomarker dynamics on the aging trajectory, we performed a first local sensitivity analysis on the (Chamberland et al., 2024) model; as such no other studies on this model are available for a direct comparison, including based on the previous (Hao and Friedman, 2016) model. Our present work focuses on local sensitivity analysis, which, in contrast to global sensitivity analysis, does not require sampling parameters from a distribution but instead relies on local perturbations around a default parameter set. This approach was chosen to enable a detailed investigation of the individual and synergistic effects of parameters on specific outputs. This includes one-at-a-time variations, relative change assessments and synergy analysis. These approaches allowed us to quantify the individual and interactive effects of parameters on neuronal count and Aβ, tau concentrations.

### 2.2 Model description

The model of Chamberland et al. (2024) consists of an ODE system of 19 variables that describes the temporal evolution of entities important in brain health in aging. These include the number of neurons, astrocytes, microglia, as well as the concentrations of amyloid beta plaques and tau neurofibrillary tangles. The relations between the entities of the model were established according to expert opinion, and the parameter values were chosen according to published experimental data from several sources. Hence, for this work we used the same equations, initial conditions, and parameters as those published in the work of Chamberland et al. (2024), with the only modification relating to the strength of the effect of APOE4 on *Aβ* plaque formation, now in line with data from Yamazaki et al. (2019). Sex (Ferretti et al., 2018) and the status of the APOE4 allele (Liu et al., 2013) are factors known to influence the risk and progression of AD, and are taken into account in the model through either their impact on the structure of the equations specifying the ODE system or on the values of parameters used in these equations. As in Chamberland et al. (2024), we therefore examined four distinct cases: women and men, both with and without the APOE4 allele. This stratification enabled us to investigate potential sex-specific and genetic differences in the model's sensitivity to parameter perturbations. The solutions of our standard model are illustrated in Figure 1. The equations specifying the model are given in Appendix A. The model and all analyses were implemented in Python, and our code is available online. The code is available at https://git.valeria.science/medics/models/sensitivity-analysis.

### 2.3 One-at-a-time sensitivity analysis (basic sensitivity analysis)

To evaluate the sensitivity of our model outcomes to individual parameter variations, we performed a One-at-a-Time (OAT) to assess how the value of each parameter affected model outcomes. We first performed an analysis where each of the 75 parameters was independently modified by +5%, +10%, and –10% from the baseline values. For each parameter, we generated a separate figure that shows the results corresponding to these perturbations, illustrating the impact on key biomarkers. This approach allows us to observe the direct influence of each parameter on the outcomes of interest.

### 2.4 Single parameter sensitivity analysis (relative change)

We computed the sensitivity of outcomes of interest at 80 years of age in response to changes in the value of a single parameter. Our outcome measures were the neuronal count (*N*) and *Aβ*/τ protein concentrations. The parameters were individually modified by +/–10%, and the mean relative change in outcome served as a measure of sensitivity. The value of 10% was chosen to reflect realistic biological variability. The sensitivity results were classified to identify the most influential parameters affecting the level of *Aβ*, *N*, and τ. The most important parameters identified by this approach were thus different depending on the outcome considered. We considered sensitivity results in absolute values because we wanted to identify the important parameters independently of whether they have a positive or negative impact on outcomes. Specifically, we computed:


$$\text{Relative Change}=\frac{\left|\text{Modified Outcome}-\text{Original Outcome}\right|}{\left|\text{Original Outcome}\right|}.$$

Dividing by the original output value yielded relative sensitivities that made the results insensitive to the absolute value of the standard outcome. As our model contains 75 distinct parameters, we conducted 300 sensitivity computations, one for each APOE/Sex conformation.

### 2.5 Parameter-pair sensitivity analysis

In our second series of experiments, we grouped parameters according to their pathways of action. We perturbed pairs of parameters belonging to different groups, simultaneously changing their values by 10%. We evaluated the outcomes changes resulting from this combined parameter change compared to the sum of the outcome changes resulting from the individual parameter change; in other words, their synergy. Suppose that we consider two parameters *A* and *B*, we define the synergy of *A* and *B* as follows:


$$\begin{array}{l} \text{Synergy}(A,B)=\\ \;[\left(\text{Outcome}(A+\Delta A,B+\Delta B)-\text{Outcome}(A,B)\right)\\ -\;\left(\text{Outcome}(A+\Delta A,B)-\text{Outcome}(A,B)\right)\\ -\;\left(\text{Outcome}(A,B+\Delta B)-\text{Outcome}(A,B)\right)]\\ /\text{Outcome}(A,B) \end{array}$$

A positive synergy (synergy > 0) implied a combined effect greater than individual effects, while a negative synergy (synergy < 0) suggested antagonism. It should be noted that synergy analysis requires *N*_*par*_ × (*N*_*par*_ − 1)/2 synergy calculations, where *N*_*par*_ is the number of model parameters for each population. Since we considered 75 parameters in 4 APOE/ Sex experimental conditions, we had to compute 11100 distinct synergy values. The large amount of data generated by these analyses required a selection for graphical display. We chose, for any two groups of parameters, to display the largest synergy in absolute value for any pairs of parameters belonging to these groups. Our figures thus illustrate the strength of interactions between different pathways of action in the model.

## 3 Results

### 3.1 One-at-a-time sensitivity analysis

**Figure 2:**
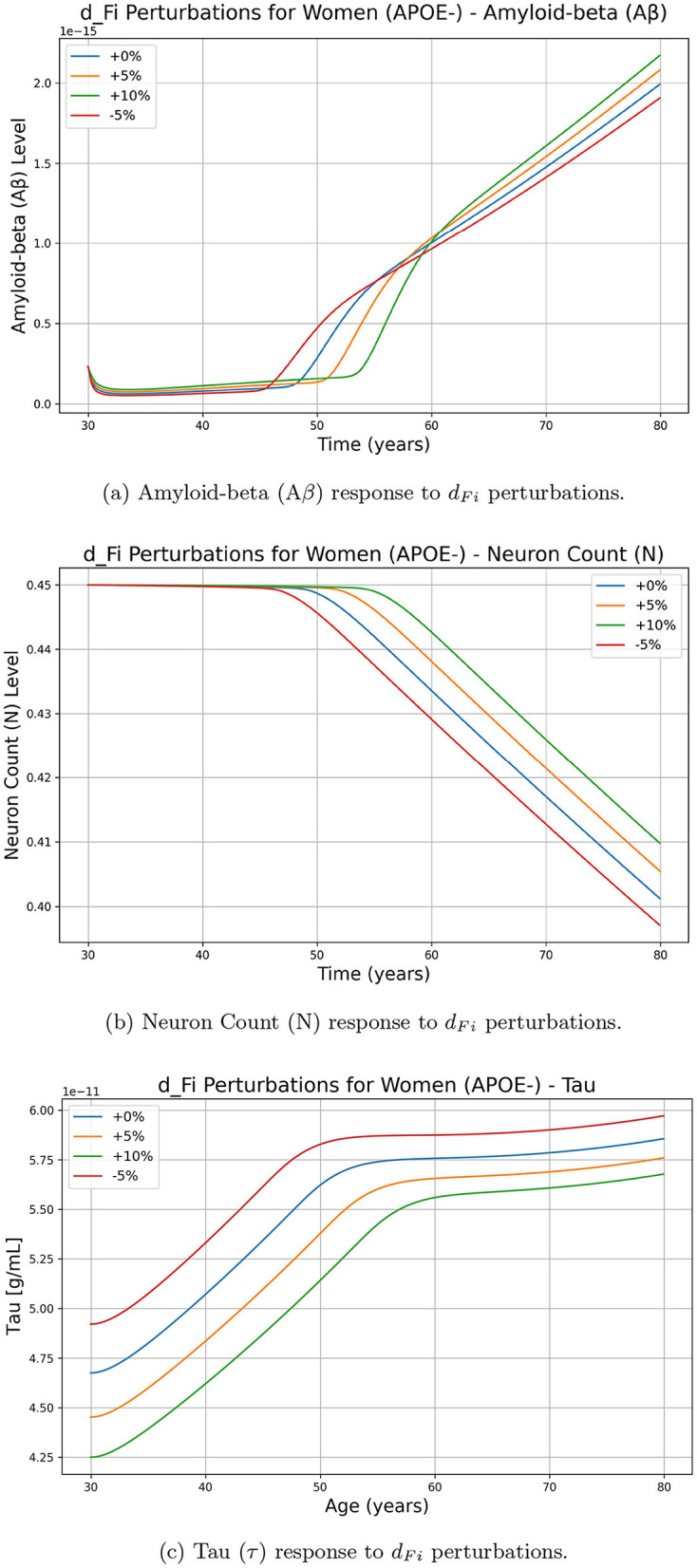
Effect of *d*_*Fi*_ perturbations on Alzheimer's-related biomarkers amyloid-beta (Aβ), neuron count (N), and tau protein (τ) over time for women with APOE-. The perturbations include 0% (baseline), +5%, +10%, and -5% changes in *d*_*Fi*_. **(a)** Amyloid-beta (Aβ) response to *d*_*Fi*_ perturbations. **(b)** Neuron Count (N) response to *d*_*Fi*_ perturbations. **(c)** Tau (τ) response to *d*_*Fi*_ perturbations.

To assess the impact of parameters on key biomarkers, we analyzed the effects of perturbations on each parameter (for example *d*_*Fi*_ the rate of intracellular degradation of NFT or lambda_Gtau_ for the rate of tau creation by GSK3) on amyloid beta (Aβ), neuron count (N) and tau protein (τ). While similar computations were performed for all parameters, we chose to illustrate the impact of *d*_*Fi*_ and lambda_Gtau_ because of their importance in the model. Figure 2 presents the temporal evolution of biomarkers of interest under different perturbations of the chosen parameters, demonstrating how variations in these parameters influence the progression of the disease in women with APOE-. Figure 2 shows the effect of increasing and decreasing *d*_*Fi*_ on (Aβ), N, and Tau concentrations over time. The results indicate that an increase in *d*_*Fi*_ (+5%, +10%) leads to a higher Aβ concentration, suggesting a positive effect on Aβ accumulation. Meanwhile, the neuron count (N) decreased more rapidly for higher values of *d*_*Fi*_, indicating a negative effect on neuronal survival. Tau protein levels increase with increasing *d*_*Fi*_, further supporting a positive effect on Tau concentration. Conversely, decreasing *d*_*Fi*_ by -5% results in lower levels of (Aβ) and Tau, with a slower decline in N, indicating a protective effect. These findings suggest that *d*_*Fi*_ plays a crucial role in accelerating disease progression by enhancing amyloid-beta accumulation and tau pathology while reducing neuronal viability.

**Figure 3:**
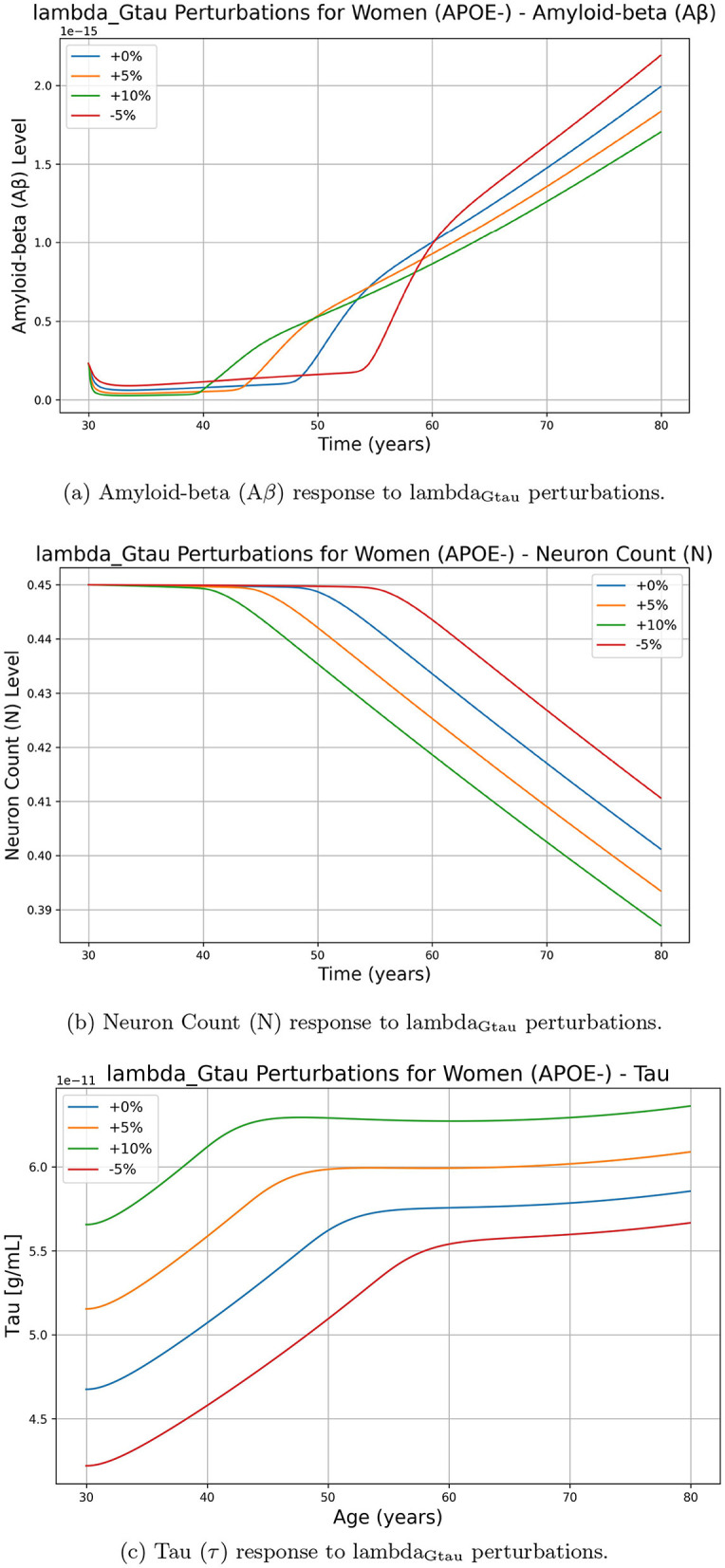
Effect of lambda_Gtau_ perturbations on Alzheimer's-related biomarkers amyloid-beta (Aβ), neuron count (N), and tau protein (τ) over time for women with APOE-. The perturbations include 0% (baseline), +5%, +10%, and -5% changes in lambda_Gtau_. **(a)** Amyloid-beta (Aβ) response to lambda_Gtau_ perturbations. **(b)** Neuron Count (N) response to lambda_Gtau_ perturbations. **(c)** Tau (τ) response to lambda_Gtau_ perturbations.

On the other hand, Figure 3 illustrates the effects of perturbing lambda_Gtau_ on Aβ, tau, and neuron(N) levels. Unlike *d*_*Fi*_, changes in lambda_Gtau_ do not lead to a straightforward increase in Aβ accumulation. Instead, a higher lambda_Gtau_ (+5%, +10%) results in a complex nonlinear response in Aβ, where the increase is not as pronounced or consistent as with *d*_*Fi*_. Although both parameters impact neuronal decline, as we increase *d*_*Fi*_ neuron decline slows down, while if we increase parameter lambda_Gtau_ values neuron decline increases rapidly. Although we show results for the women APOE negative population, similar trends were observed for other groups.

### 3.2 Single-parameter sensitivity analysis

**Figure 4:**
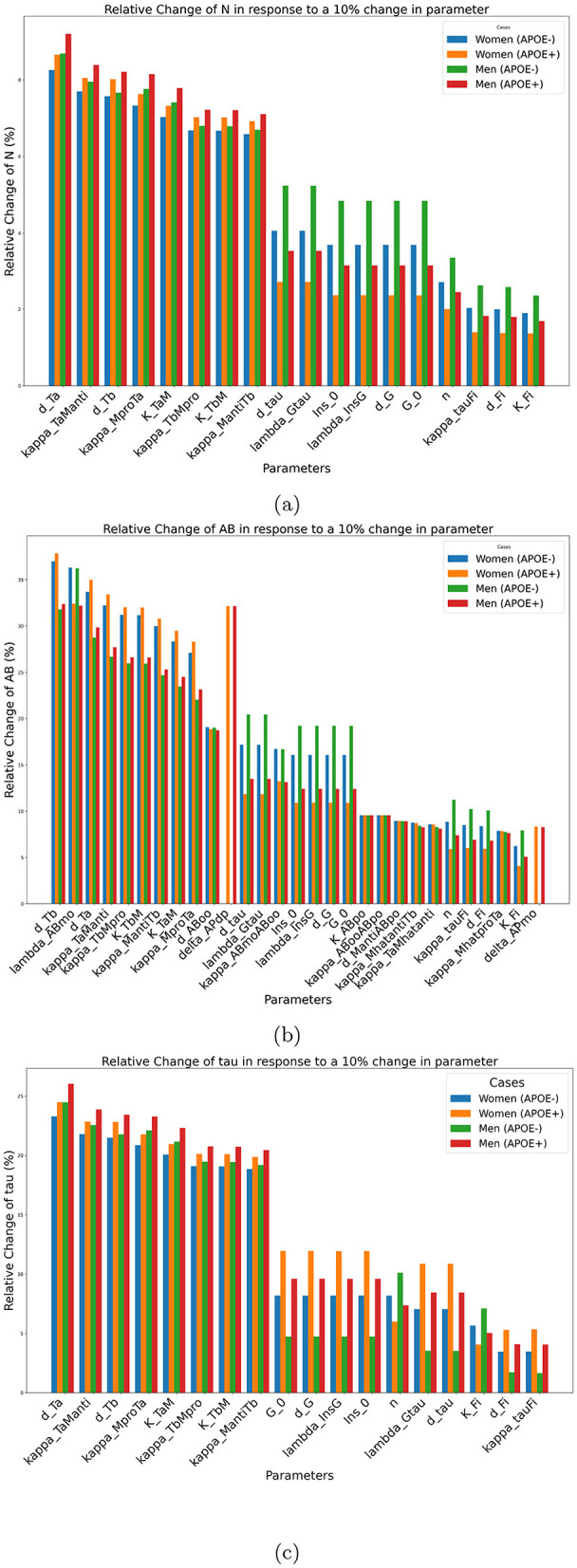
Sensitivity of outcomes of interest to individual parameters for biomarkers. **(a)** N, **(b)** *Aβ*, **(c)** Tau at age 80 years in response to a 10% change in individual parameter values. These plots illustrate the sensitivity of each outcome to parameter perturbations, highlighting their potential impact on disease progression.

The results of the impact of the changes in a single parameter on our three results are shown in Figure 4.

The most important factor identified by this analysis relating to neuronal density and τ concentration is *d*_*Ta*_. This parameter corresponds to the decay rate of the tumor necrosis factor alpha. Increasing this factor leads to a decrease in the concentration of tumor necrosis alpha, which is in turn beneficial for neuron survival. As the role of metabolism is often neglected in mathematical models of AD, it is interesting to observe that parameters related to insulin and glucose regulation, such as *Ins*_0_, the baseline insulin concentration, λ_InsG_, the glycogen synthase kinase 3 (GSK-3) dynamics *G*_0_, the baseline concentration of GSK-3 and *d*_*G*_, the autonomous decay rate of GSK-3, have significant impacts with respect to both neuronal density and tau concentration.

Regarding Aβ plaque concentration, the most important important parameter is *d*_*Tb*_, which corresponds to the decay rate of the transforming growth factor beta (*T*_β_). This factor is involved in the activation of microglia and macrophages. A higher concentration of *T*_β_ will lead to a higher proportion of anti-inflammatory microglia and macrophages, which in turn play a role in increasing amyloid beta plaque clearance. It is somewhat surprising that the parameter that has the greatest impact on amyloid beta concentration does so through an indirect pathway. Through its influence on Aβ, the parameter *d*_*Tb*_ has an even more indirect impact on neuronal density. Note that this effect is still important as *d*_*Tb*_ ranks third with respect to neural density sensitivity. This first investigation also revealed that Aβ sensitivity is larger than neuronal density or the tau concentration. This implies that relatively small perturbations of the model can lead to significant Aβ accumulation.

We observe furthermore that this single-parameter sensitivity is affected by sex and APOE status, and that this is true for all parameters. Due to the way the model is constructed, the parameters that quantify the effect of the APOE gene, such as delta_APdP_ and delta_APmo_ had an effect only for the positive populations of APOE. For each outcome, the parameters are ranked according to their average effect on the four investigated groups (men, women, positive, and negative APOE).

### 3.3 Parameter-pair sensitivity analysis

**Figure 5:**
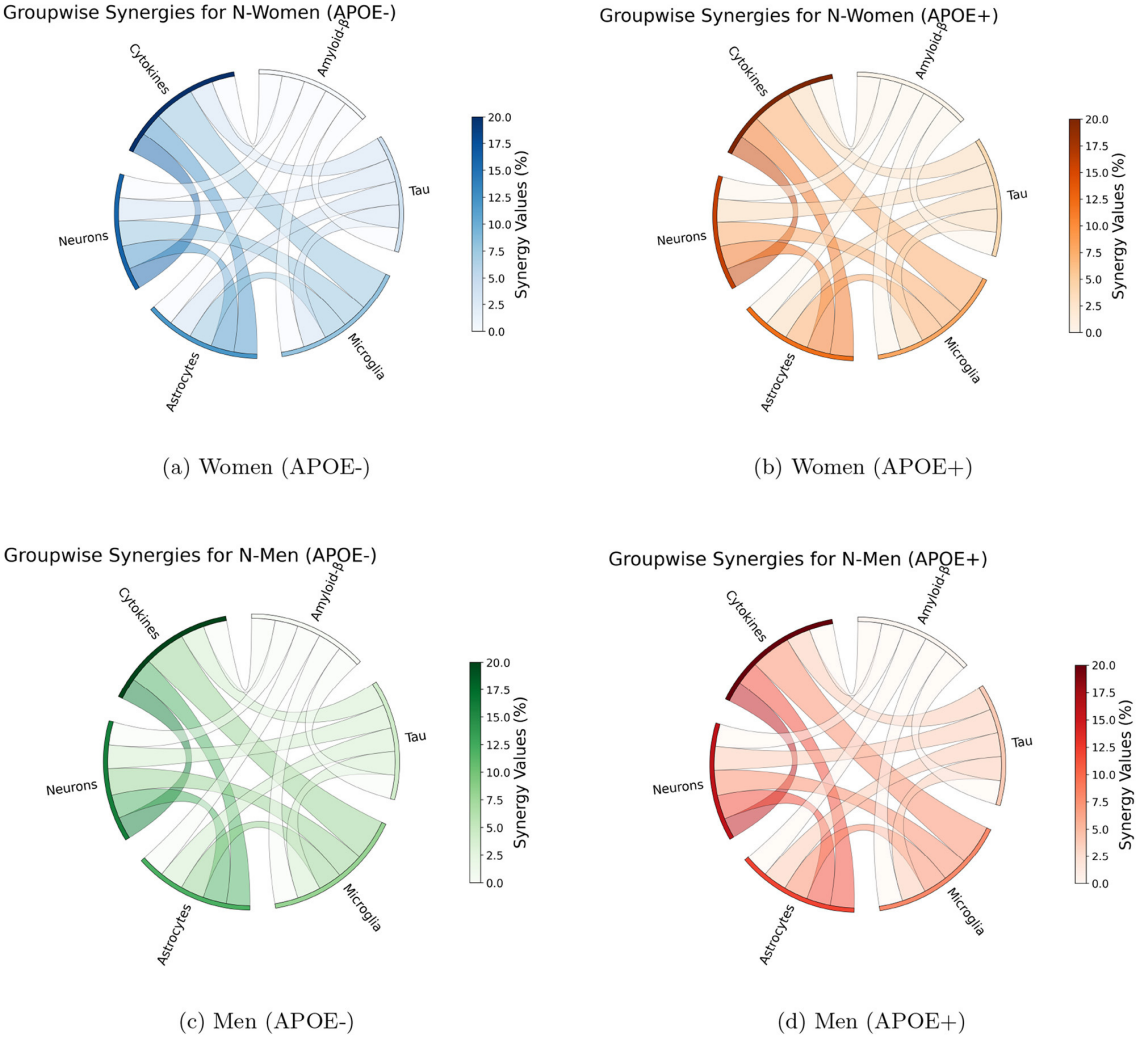
Synergy analysis of group-wise parameter interactions for Alzheimer's disease biomarker across different demographic groups. Each subfigure represents a demographic group: **(a)** Women (APOE-), **(b)** Women (APOE+), **(c)** Men (APOE-), and **(d)** Men (APOE+). Synergy values are calculated as the difference between the combined effect of interacting parameters and the sum of their individual effects. Maximal synergies between parameters belonging to given groups are shown. Arc color indicates interaction magnitude, with darker colors representing higher magnitudes.

Parameter-pair synergy analyzes with respect to neural count at 80 years of age unveiled significant interactions between the effect of parameters, suggesting that the effect of combination therapy could be more important than the sum of individual effects. In particular, strong synergies were observed between parameters that act directly on neural survival and those that affect cytokine concentrations Figure 5. Interactions were also observed between parameters that affect the density of astrocytes and those involved in the density of cytokines or directly impact the survival of the neuron. When considered group-wise, parameters involved in astrocytes, neurons, and cytokines have the strongest interactions with the parameters of other groups with a strength of interactions reaching 20%.

We identified case-specific differences in synergy patterns across the four APOE/Sex conformations. Although similar trends were observed in the analysis of these different populations, subtle differences were also observed Table 1. In particular, absolute strength of interactions slightly stronger in APOE positive men. These differences show that the status of APOE4 has an effect on how the disease factors that work together cause AD to worsen (Yamazaki et al., 2019). This observation underscores the complexity of AD and the need for nuanced, personalized approaches.

**Table 1:** Synergy values (%) by sex, APOE status, and interaction type.

**Sex**	**APOE status**	**Synergy value (%)**	**Interaction**
Women	APOE-	18.84	Microglia–Cytokines
Women	APOE+	19.87	Microglia–Cytokines
Men	APOE+	**20.32**	Microglia–Cytokines
Men	APOE-	19.00	Microglia–Cytokines

Synergy values shown are for the interaction between microglia and cytokines. Among the different groups analyzed, this interaction consistently exhibits the highest synergy across all sex and APOE status categories. Bold values indicate the highest synergy values (%) within each subgroup (sex, APOE status, and interaction type).

## 4 Discussion

Mathematical models of AD frequently focus on the accumulation of harmful proteins such as τ and *Aβ*. Some employ sophisticated mathematical techniques to forecast the patterns of spread in brain geometries peculiar to individual patients (Corti, 2024), while others examine the interaction between various proteins and neuronal dynamics (Moravveji et al., 2024; Bertsch et al., 2020). However, without a comprehensive analysis of how the various inputs of a model influence its outcome, mathematical models are of little use. Thus, sensitivity analysis is essential to understand the influence of different parameters on the model output (Corti, 2024; Sysoev, 2023; Moravveji et al., 2024). Our goal here was to identify the most important parameters of the multifactorial knowledge-based AD model developed by Chamberland et al. (2024).

### 4.1 Identification of critical parameters

The sensitivity analysis revealed which parameters play an important role with respect to specific outcomes of interest (*Aβ*, *N*, and τ). It is interesting to observe that some parameters have an important impact on *Aβ* (λ_*Aβmo*_, *AP*, *d*_*Aβoo*_, kappa_AβmoAβoo_) but not on neuronal count. This suggests that treatments targeting *Aβ* specifically, even if they succeeded in preventing *Aβ* plaque accumulation, are likely to fail to prevent neuronal loss. Sensitivity analysis also revealed that some of the parameters involved in the insulin pathway have a significant impact on neurodegeneration. This suggests paying more attention to metabolic-related issues, which have been neglected in many experimental and model studies. The one-at-a-time sensitivity analysis and the relative change analysis showed that the concentration of *Aβ* at 80 years old is much more sensitive to parameter variations than the neuronal count or tau concentration. This implies that, in the (Chamberland et al., 2024) model, limited (e.g., 10%) changes in parameter values can have a huge impact on the presence of *Aβ* at the end of life; and that the relationships between *Aβ* concentration and parameters are strongly nonlinear. In other words, *Aβ* does not vary smoothly in response to changes in parameter values, but rather exhibits sharp monotonic transitions.

This can be explained by the observation that while under standard parameter values the model leads to a very small Aβ concentration, some parameter configurations corresponding to biologically pathological situations lead to large Aβ accumulation.

This suggests a non-linear response, where small variations in key parameters can drastically influence Aβ levels. An explanation might be that when the amyloid plaque starts accumulating, this triggers a positive feedback loop that accelerates further deposition. This would create a kind of all-or-nothing scenario in which either very little or a large quantity of amyloid beta plaques are present at the end of life.

The observed differences in the sensitivity results by sex and APOE4 status emphasize the importance of personalized approaches in AD treatment. Our findings indicate that tailoring therapies based on these factors could enhance efficacy and shift away from a universal approach to AD management.

### 4.2 APOE4 impact on Aβ dynamics

The parameter δ_*APdp*_ describes the impact of the APOE4 allele on the rate of degradation of amyloid beta42 plaques by anti-inflammatory macrophages and microglia. Although Gonneaud et al. (2016) and Mishra et al. (2018) reported an annual increase in amyloid deposition 5%, this refers to the general accumulation rate rather than the specific degradation rate by glial cells in carriers of APOE4. Furthermore, Xia et al. (2024) suggest that APOE4 primarily influences the aggregation of monomers and oligomers rather than directly affecting plaque degradation.

In our main model, we initially set δ_*APdp*_ = −0.75 (Chamberland et al., 2024), meaning that non-APOE4 carriers were more likely to have plaque removed by a factor (4:1) (see Figure 1). However, our sensitivity analysis showed that the levels of Aβ were 100 times higher in the APOE group than in the APOE + group (10E-14 mol/L vs. 10E-16 mol/L; see Figure 4a).

This substantial variability in Aβ levels can be attributed to parameter uncertainties and underscores potential differences between the negative and positive groups of APOE, which could contribute to different risk profiles for AD. To further investigate this, we tested the impact of modifying δ_*APdp*_. In particular, reducing it from –0.75 to –0.5 resulted in approximately a 10-fold decrease in plaque concentration at 80 years of age.

Given these considerations, the precise quantification of δ_*APdp*_ remains a challenge. However, the association between APOE4 and increased amyloid accumulation (Liu et al., 2013) suggests a potential reduction in plaque clearance efficiency. The effect is likely more subtle than a reduction in the degradation rate 50% or 75%. A more conservative estimate might range from –0.2 to –0.3, representing a 20% to 30% reduction in the degradation rate for APOE4 carriers. However, this estimate requires validation against the experimental data.

These findings present us with an intriguing dichotomy in interpretation. On the one hand, we must consider the possibility that our model may have overestimated the influence of APOE, potentially necessitating a recalibration of our parameters. This interpretation would suggest that, while APOE4 undoubtedly plays a significant role in AD pathogenesis, its impact on Aβ dynamics might be less extreme than our current model indicates. However, the predictions of our model might accurately reflect the substantial impact of APOE on Aβ plaque formation and clearance of Aβ, capturing a fundamental aspect of the progression of AD that has been underappreciated in previous mathematical modeling studies.

Recent research supports the latter interpretation (Liu et al., 2023) demonstrated that APOE4 significantly impairs the ability of microglia to clear Aβ, which could explain the dramatic differences observed in our model. Furthermore, Tachibana et al. (2019) found that APOE4 improves Aβ aggregation and impedes its clearance, in line with the predictions of our model.

The impact of APOE4 on Aβ dynamics is further complicated by its influence on microglial and macrophage function. The model equations we made for microglia Equations (11–13) and macrophages Equations (14, 15) show that turning these cells on and off is a key part of removing Aβ. APOE4 has been shown to alter the balance between pro-inflammatory and anti-inflammatory states of these cells (Krasemann et al., 2017), which could exacerbate Aβ accumulation.

In addition, the interaction between APOE4 and Aβ can influence the activation of microglia and macrophages. The term $\kappa_{{A\beta }_{o}^{o}M}\frac{{A\beta }_{o}^{o}}{{A\beta }_{o}^{o}+K_{{A\beta }_{o}^{o}}}$ in Equation A7 represents the activation of microglia by Aβ oligomers. APOE4 has been shown to enhance this activation (Zhu et al., 2012), potentially leading to a more pronounced inflammatory response and altered Aβ clearance dynamics.

These findings highlight the critical role of APOE in AD pathogenesis and suggest that our model, despite its apparent extremity, may capture a fundamental aspect of APOE4's impact on Aβ dynamics. The complex interplay between APOE4, Aβ, and immune cells underscores the need for multiscale modeling approaches, as exemplified by Chamberland et al. (2024), to fully understand the disease process. However, further experimental validation is needed to confirm these computational predictions and refine our understanding of the influence of APOE on the progression of AD.

### 4.3 Implications of the synergy analysis results

Other approaches can be used to obtain an index of synergy, such as those considering isobolograms (Huang et al., 2019), but our simple implementation captures the same intuitive idea.

Our findings have far-reaching implications for AD research and treatment strategies. The strong interactions revealed by our synergistic analysis suggest that, with the right targets, combination therapies could be better at slowing the progression of AD than single-target approaches. This insight opens new avenues for drug development and clinical trials, potentially leading to more efficacious treatments for AD patients.

Moreover, the synergy pattern observed for N indicates that multi-pronged treatment strategies targeting different aspects of AD pathology might be necessary for comprehensive disease management. This finding challenges the current paradigm of focusing on a single aspect of AD pathology and suggests that a more holistic approach to treatment could lead to better results.

From a modeling perspective, our synergy analysis provides valuable insight to refine our AD model. Parameters involved in strong synergistic or antagonistic interactions may require more detailed modeling or further experimental investigation to better understand their roles in the progression of AD. This iterative process of model refinement and experimental validation can lead to more accurate and predictive models of AD pathology.

Lastly, our findings highlight specific parameter interactions that warrant further investigation through targeted experimental studies. These could lead to new insights into AD mechanisms and novel therapeutic approaches. By identifying these key areas for future research, our synergy analysis serves as a roadmap for upcoming studies on AD pathology and treatment.

## 5 Conclusion

Future research should focus on experimentally validating the relationships identified in our model and exploring how interventions targeting these parameters might influence the overall trajectory of neuronal decline in Alzheimer's disease. Alzheimer's disease is a multifactorial condition with a complex etiology. Studies focusing on a single factor, such as amyloid beta, have had limited success. We argue for the importance of considering an integrative approach. Computer models, such as the one we employed, offer an opportunity to test multiple factors *in silico* and are an ideal way to integrate various hypotheses.

We carried out various analyses on a mathematical model of brain aging developed by (Chamberland et al., 2024) that takes into account APOE status and sex. This model was constructed based on expert opinion and calibrated with different sources of experimental literature. A table in Appendix B presents the biological meaning and sources of the selected parameters. These parameters are identical to those used in the original (Chamberland et al., 2024) model and form part of the system of equations that connects all entities in the neurodegeneration model.

We performed a local sensitivity analysis using one-at-a-time perturbations, relative changes that revealed the most impactful parameters, as well as pairwise parameter interactions (synergy analysis) revealing nonlinear interactions between parameter effects. This analysis reveals potential synergies between treatment approaches.

Our study provided interesting insights. First, it suggests that parameters related to glucose and insulin regulation might play an important role in neurodegeneration and cognitive decline. Second, the parameters that have the most important impact on cognitive decline are not completely the same depending on sex and APOE status. This observation supports the importance of personalized treatment approaches that take this into account. It is interesting that small changes in parameter values can lead to relatively large changes in amyloid beta concentration.

In future work, we plan to perform a global sensitivity analysis by generating a virtual population by sampling the parameters and conducting a correlation analysis. This will help us better understand the relationships between different parameters and their combined effects on the progression of Alzheimer's disease. To ensure reproducibility and robustness, the analysis will also involve systematically varying initial conditions and comparing the outcomes between different cohorts. Ultimately, our goal is to refine the model and investigate how complex interactions among biological factors influence disease outcomes, while validating its accuracy and generalizability.

Although this study makes valuable contributions, we recognize that it has certain limitations. For one, some elements may be missing from the model that could influence our conclusion. In addition, we did not address the variability of parameters in a real population or whether it is possible to alter the value of a parameter through intervention.

## Data Availability

The original contributions presented in the study are included in the article/Supplementary material, further inquiries can be directed to the corresponding authors.
