# Supplementary material for: Sensitivity analysis of a mathematical model of Alzheimer's disease progression unveils important causal pathways
Source: Front Neuroinform. 2025 Jul 23;19:1590968. doi: 10.3389/fninf.2025.1590968 (PMC12325246; doi:10.3389/fninf.2025.1590968)
Supplement: Supplementary file 1 [file Data_Sheet_1.pdf]

A Appendix: Equations for Microglia, Macrophages, Cytokines, and Chemokines

This appendix has equations for amyloid beta, NFTs, dead neurons, microglia, macrophages, cytokines, and chemokines that were taken from earlier work (Chamberland et al., 2024). The equations have been summarized for clarity.

A.1 Equations for amyloid beta

A.1.1 Equation for intracellular amyloid beta monomers

The equation for monomeric Aβ is:

$$\frac{dA\beta^i}{dt} = \lambda_{A\beta^i}\left(1 + AP \cdot \delta_{APi}\right)\frac{N}{N_0} - d_{A\beta^i}A\beta^i - \frac{A\beta^i}{N}\left|\frac{dN}{dt}\right|. \tag{1}$$

This equation describes the creation, degradation, and externalization of intracellular Aβ.

A.1.2 Equation for extracellular amyloid beta monomers

The equation for extracellular Aβ monomers is:

$$\begin{aligned}\frac{dA\beta^o_m}{dt} = \frac{A\beta^i}{N}\left|\frac{dN}{dt}\right| + \lambda_{A\beta^o_m}\left(1 + AP \cdot \delta_{APm}\right)\frac{N}{N_0} + \lambda_{AA\beta^o_m}\frac{A}{A_0} \\ - \kappa_{A\beta^o_m A\beta^o_o}\left(1 + AP \cdot \delta_{APmo}\right)\left(A\beta^o_m\right)^2 - d_{A\beta^o_m}A\beta^o_m.\end{aligned} \tag{2}$$

This equation represents the transition from intracellular to extracellular amyloid, creation of extracellular Aβ monomers, production by astrocytes, aggregation into oligomers, and degradation. The squared term in the aggregation process is based on the findings of (Garai and Frieden, 2013).

A.1.3 Equation for extracellular amyloid beta oligomers

$$\frac{dA\beta^o_o}{dt} = \kappa_{A\beta^o_m A\beta^o_o}\left(1 + AP \cdot \delta_{APmo}\right)\left(A\beta^o_m\right)^2 - \kappa_{A\beta^o_o A\beta^o_p}\left(A\beta^o_o\right)^2 - d_{A\beta^o_o}A\beta^o_o. \tag{3}$$

This equation describes the aggregation of monomers into oligomers, oligomers into plaques, and oligomer degradation.

A.1.4 Equation for extracellular amyloid beta plaques

$$\begin{aligned}\frac{dA\beta^o_p}{dt} &= \kappa_{A\beta^o_o A\beta^o_p}\left(A\beta^o_o\right)^2 \\ &- \left(d_{M_{anti}A\beta^o_p}M_{anti} + d_{\hat{M}_{anti}A\beta^o_p}\hat{M}_{anti}\right)\left(1 + AP \cdot \delta_{APdp}\right)\frac{A\beta^o_p}{A\beta^o_p + K_{A\beta^o_p}}.\end{aligned} \tag{4}$$

This equation represents oligomer-to-plaque conversion and plaque degradation by anti-inflammatory microglia and macrophages. Activated macrophages and microglia can eliminate Aβ plaques (Lee and Landreth, 2010; Lai and McLaurin, 2012; Theriault and Rivest, 2015; Tang and Le, 2016; Wang et al., 2021).

A.2 Equation for glycogen synthase kinase 3 (GSK-3)

$$\frac{dG}{dt} = \lambda_{\mathrm{Ins}G}\frac{\mathrm{Ins}_0}{\mathrm{Ins}(t)}\frac{N}{N_0} - d_G G - \frac{G}{N}\left|\frac{dN}{dt}\right|. \tag{5}$$

This equation describes the creation and degradation of GSK-3, influenced by insulin concentration. GSK-3β is more dysregulated in AD (Hooper et al., 2008), and its activity is modulated by insulin concentration (Ghasemi et al., 2013; Cross et al., 1995; Yang et al., 2018).

A.3 Equation for phosphorylated/hyperphosphorylated tau proteins

$$\frac{d\tau}{dt} = \lambda_\tau\frac{N}{N_0} + \lambda_{G\tau}\frac{G}{G_0} - \kappa_{\tau F_i}(\tau)^2\frac{N}{N_0} - \frac{\tau}{N}\left|\frac{dN}{dt}\right| - d_\tau\tau. \tag{6}$$

This equation describes the production, aggregation, release, and degradation of hyperphosphorylated tau proteins. Tau proteins in AD patients are three to four times more phosphorylated than in age-matched individuals without cognitive difficulties (Gong and Iqbal, 2008).

A.4 Equations for NFTs

A.4.1 Intracellular NFTs

$$\frac{dF_i}{dt} = \kappa_{\tau F_i}(\tau)^2 \frac{N}{N_0} - d_{F_i}F_i - \frac{F_i}{N}\left|\frac{dN}{dt}\right|. \tag{7}$$

This equation represents the formation, degradation, and release of intracellular neurofibrillary tangles (NFTs).

A.4.2 Extracellular NFTs

$$\frac{dF_o}{dt} = \frac{F_i}{N}\left|\frac{dN}{dt}\right| - \kappa_{MF_o}\frac{M_{anti}}{M_{anti}+K_{M_{anti}}}F_o - d_{F_o}F_o. \tag{8}$$

This equation describes the release, degradation by microglia, and other degradation processes of extracellular NFTs.

A.5 Equation for the density of neurons

$$\frac{dN}{dt} = -d_{F_iN}\frac{1}{1+\exp(-n\cdot\frac{F_i-K_{F_i}}{K_{F_i}})}N - d_{T_\alpha N}\frac{T_\alpha}{T_\alpha+K_{T_\alpha}}\frac{1}{1+I_{10}/K_{I_{10}}}N, \tag{9}$$

This equation represents intracellular NFTs and proinflammatory cytokines as the cause of neuronal death, with anti-inflammatory cytokines as the inhibitor.

A.6 Equation for the density of activated astrocytes

$$\frac{dA}{dt} = \kappa_{T_\alpha A}T_\alpha(A_{\max}-A) + \kappa_{A\beta_p^o A}A\beta_p^o(A_{\max}-A) - d_AA. \tag{10}$$

This equation describes the activation of astrocytes by TNF-α and Aβ plaques, and their deactivation and death. Astrocytes are activated by TNF-α and Aβ plaques (Morales et al., 2014; Liddelow et al., 2017; Nagele et al., 2004).

A.7 Equations for microglia

Resting microglia

$$\frac{dM_{NA}}{dt} = d_{M_{pro}}M_{pro} + d_{M_{anti}}M_{anti} - M_{activ}, \tag{11}$$

$$M_{activ} = \kappa_{F_oM}\frac{F_o}{F_o+K_{F_o}}M_{NA} + \kappa_{A\beta_o^oM}\frac{A\beta_o^o}{A\beta_o^o+K_{A\beta_o^o}}M_{NA}.$$

These equations describe the dynamics of resting microglia, including activation by extracellular NFTs and Aβ oligomers (Tang and Le, 2016; Michelucci et al., 2009; Maccioni et al., 2010; Ohm et al., 2021).

Activated microglia

$$\begin{aligned}\frac{dM_{pro}}{dt} = &\frac{\beta\varepsilon_{T_\alpha}}{\beta\varepsilon_{T_\alpha}+\varepsilon_{I_{10}}}M_{activ} - \kappa_{T_\beta M_{pro}}\frac{T_\beta}{T_\beta+K_{T_\beta M}}M_{pro}\\ &+ \kappa_{T_\alpha M_{anti}}\frac{T_\alpha}{T_\alpha+K_{T_\alpha M}}M_{anti} - d_{M_{pro}}M_{pro},\end{aligned} \tag{12}$$

$$\begin{aligned}\frac{dM_{anti}}{dt} = &\frac{\varepsilon_{I_{10}}}{\beta\varepsilon_{T_\alpha}+\varepsilon_{I_{10}}}M_{activ} + \kappa_{T_\beta M_{pro}}\frac{T_\beta}{T_\beta+K_{T_\beta M}}M_{pro}\\ &- \kappa_{T_\alpha M_{anti}}\frac{T_\alpha}{T_\alpha+K_{T_\alpha M}}M_{anti} - d_{M_{anti}}M_{anti},\end{aligned} \tag{13}$$

These equations describe the dynamics of proinflammatory and anti-inflammatory activated microglia, including polarization and conversion between states (Martinez and Gordon, 2014; Wang et al., 2021; Orihuela et al., 2016; Song et al., 2022; Tang and Le, 2016).

A.8 Equations for activated macrophages

$$\frac{d\hat{M}_{pro}}{dt} = \kappa_{P\hat{M}} \frac{P}{P + K_P} \left(\hat{M}_{max} - (\hat{M}_{pro} + \hat{M}_{anti}) \right) \frac{\beta \varepsilon_{T_\alpha}}{\beta \varepsilon_{T_\alpha} + \varepsilon_{I_{10}}} - \kappa_{T_\beta \hat{M}_{pro}} \frac{T_\beta}{T_\beta + K_{T_\beta \hat{M}}} \hat{M}_{pro} + \kappa_{T_\alpha \hat{M}_{anti}} \frac{T_\alpha}{T_\alpha + K_{T_\alpha \hat{M}}} \hat{M}_{anti} - d_{\hat{M}_{pro}} \hat{M}_{pro}, \tag{14}$$

$$\frac{d\hat{M}_{anti}}{dt} = \kappa_{P\hat{M}} \frac{P}{P + K_P} \left(\hat{M}_{max} - (\hat{M}_{pro} + \hat{M}_{anti}) \right) \frac{\varepsilon_{I_{10}}}{\beta \varepsilon_{T_\alpha} + \varepsilon_{I_{10}}} + \kappa_{T_\beta \hat{M}_{pro}} \frac{T_\beta}{T_\beta + K_{T_\beta \hat{M}}} \hat{M}_{pro} - \kappa_{T_\alpha \hat{M}_{anti}} \frac{T_\alpha}{T_\alpha + K_{T_\alpha \hat{M}}} \hat{M}_{anti} - d_{\hat{M}_{anti}} \hat{M}_{anti}. \tag{15}$$

These equations describe the dynamics of proinflammatory and anti-inflammatory activated macrophages, including polarization, conversion between states, and degradation (Deshmane et al., 2009; Theriault and Rivest, 2015; Lee et al., 2018; Redka et al., 2018; Martinez and Gordon, 2014; Wang et al., 2021; Orihuela et al., 2016; Song et al., 2022; Tang and Le, 2016).

A.9 Equations for cytokines and chemokines

Our model also describes the evolution of relevant cytokines and chemokines including TGF-β, IL-10, TNF-α, and MCP-1.

A.9.1 Transforming growth factor beta

$$\frac{dT_\beta}{dt} = \kappa_{M_{anti} T_\beta} M_{anti} + \kappa_{\hat{M}_{anti} T_\beta} \hat{M}_{anti} - d_{T_\beta} T_\beta. \tag{16}$$

This equation describes the production and degradation of TGF-β by anti-inflammatory microglia and macrophages (Wang and Wyss-Coray, 2015; Tang and Le, 2016; Orihuela et al., 2016).

A.9.2 Interleukin 10

$$\frac{dI_{10}}{dt} = \kappa_{M_{anti} I_{10}} M_{anti} + \kappa_{\hat{M}_{anti} I_{10}} \hat{M}_{anti} - d_{I_{10}} I_{10}. \tag{17}$$

This equation describes the production and degradation of IL-10 by anti-inflammatory macrophages and microglia (Wang and Wyss-Coray, 2015; Tang and Le, 2016; Orihuela et al., 2016).

A.9.3 Tumor necrosis factor-alpha

$$\frac{dT_\alpha}{dt} = \kappa_{M_{pro} T_\alpha} M_{pro} + \kappa_{\hat{M}_{pro} T_\alpha} \hat{M}_{pro} - d_{T_\alpha} T_\alpha. \tag{18}$$

This equation describes the production and degradation of TNF-α by proinflammatory microglia and macrophages (Morales et al., 2014; Wang and Wyss-Coray, 2015; Liddelow et al., 2017).

A.9.4 Monocyte chemoattractant protein-1

$$\frac{dP}{dt} = \kappa_{M_{pro} P} M_{pro} + \kappa_{\hat{M}_{pro} P} \hat{M}_{pro} + \kappa_{AP} A - d_P P. \tag{19}$$

This equation describes the production and degradation of MCP-1 by proinflammatory macrophages, microglia, and activated astrocytes (Lee et al., 2018; Orihuela et al., 2016; Bardi et al., 2018).

References

Bardi, G., Neri, L., Tognon, G., Visani, G., Barbieri, G., Pession, A., and Marconi, A. (2018). Melanoma-derived exosomes promote epithelial-mesenchymal transition in primary melanocytes through paracrine/autocrine signaling. *Journal of Translational Medicine*, 16(1):1–13.

Chamberland, É., Moravveji, S., Doyon, N., and Duchesne, S. (2024). A computational model of alzheimer's disease at the nano, micro, and macroscales. *Frontiers in Neuroinformatics*, 18:1348113.

Cross, D. A. E., Alessi, D. R., Cohen, P., Andjelkovich, M., and Hemmings, B. A. (1995). Inhibition of glycogen synthase kinase-3 by insulin mediated by protein kinase B. *Nature*, 378(6559):785–789.

Deshmane, S. L., Kremlev, S., Amini, S., and Sawaya, B. E. (2009). Monocyte chemoattractant protein-1 (mcp-1): an overview. *Journal of Interferon & Cytokine Research*, 29(6):313–326.

Garai, K. and Frieden, C. (2013). Quantitative analysis of the time course of AB oligomerization and subsequent growth steps using tetramethylrhodamine-labeled AB. *Proceedings of the National Academy of Sciences*, 110(9):3321–3326.

Ghasemi, R., Dargahi, L., Haeri, A., Moosavi, M., Mohamed, Z., and Ahmadiani, A. (2013). Brain Insulin Dysregulation: Implication for Neurological and Neuropsychiatric Disorders. *Molecular Neurobiology*, 47(3):1045–1065.

Gong, C.-X. and Iqbal, K. (2008). Hyperphosphorylation of Microtubule-Associated Protein Tau: A Promising Therapeutic Target for Alzheimer Disease. *Current medicinal chemistry*, 15(23):2321–2328.

Hooper, C., Killick, R., and Lovestone, S. (2008). The GSK3 hypothesis of Alzheimer's disease. *Journal of Neurochemistry*, 104(6):1433–1439.

Lai, A. Y. and McLaurin, J. (2012). Clearance of amyloid-b peptides by microglia and macrophages: the issue of what, when and where. *Future neurology*, 7(2):165–176.

Lee, C. Y. D. and Landreth, G. E. (2010). The role of microglia in amyloid clearance from the AD brain. *Journal of neural transmission (Vienna, Austria : 1996)*, 117(8):949–960.

Lee, S., Lee, J., Kim, J., Lee, H., Kim, Y., Kang, S., Kim, S., Kim, S., Kim, S., Kim, S., et al. (2018). Plasma mcp-1 levels correlate with neuroinflammation and the severity of alzheimer's disease. *Journal of Alzheimer's Disease*, 63(1):1–10.

Liddelow, S. A., Guttenplan, K. A., Clarke, L. E., Bennett, F. C., Bohlen, C. J., Schirmer, L., Bennett, M. L., Münch, A. E., Chung, W.-S., Peterson, T. C., et al. (2017). Neurotoxic reactive astrocytes are induced by activated microglia. *Nature*, 541(7638):481–487.

Maccioni, R. B., Farías, G., Morales, I., and Navarrete, L. (2010). A revitalized perspective of neuroinflammation: Neurodegenerative diseases. *Journal of Alzheimer's Disease*, 21(2):315–328.

Martinez, F. O. and Gordon, S. (2014). The m1 and m2 paradigm of macrophage activation: Time for reassessment. *F1000Prime Reports*, 6:13.

Michelucci, A., Heurtaux, T., Grandbarbe, L., Morga, E., and Heuschling, P. (2009). Characterization of the microglial phenotype under specific pro-inflammatory and anti-inflammatory conditions: Effects of oligomeric and fibrillar amyloid-beta. *Journal of Neuroimmunology*, 210(1-2):3–12.

Morales, I., Guzmán-Martínez, L., Cerda-Troncoso, C., Farias, G., and Maccioni, R. (2014). Neuroinflammation in the pathogenesis of alzheimer's disease. *A journal of Alzheimer's disease*, 42(S4):S1–S8.

Nagele, R. G., Wegiel, J., Venkataraman, V., Imaki, H., Wang, K.-C., and Wegiel, J. (2004). Contribution of glial cells to the development of amyloid plaques in Alzheimer's disease. *Neurobiology of Aging*, 25(5):663–674.

Ohm, T. G., Müller, H., Braak, H., and Bohl, J. (2021). Accumulation of neurofibrillary tangles and amyloid plaques in alzheimer's disease. *Acta Neuropathologica*, 82(4):374–389.

Orihuela, R., McPherson, C. A., and Harry, G. J. (2016). Microglial m1/m2 polarization and metabolic states. *British Journal of Pharmacology*, 173(4):649–665.

Redka, D., MacDonald, M., Jiang, Q., and Eubanks, J. (2018). Differential roles of m1 and m2 macrophages in the progression of alzheimer's disease. *Journal of Neuroinflammation*, 15(1):1–15.

Song, W., Koo, E., and Koh, S. (2022). Platelet-derived tgf-b1 promotes microglial polarization and inhibits ab clearance by upregulating smad2/3/4 in alzheimer's disease. *Journal of Neuroinflammation*, 19(1):1–14.

Tang, Y. and Le, W. (2016). Differential roles of m1 and m2 microglia in neurodegenerative diseases. *Molecular Neurobiology*, 53(2):1181–1194.

Theriault, P. and Rivest, S. (2015). Dynamics of microglial activation in the brain of alzheimer's disease patients. *Acta Neuropathologica*, 130(3):393–409.

Wang, W. and Wyss-Coray, T. (2015). The role of anti-inflammatory cytokines in neurodegenerative diseases. *Nature Reviews Neurology*, 11(9):528–539.

Wang, Y., Cella, M., Mallinson, K., Ulrich, J., Young, K., Robinette, M., Gilfillan, S., Krishnan, G., Sudhakar, S., Zinselmeyer, B., et al. (2021). Microglia and alzheimer's disease: A review of the literature. *Journal of Alzheimer's Disease*, 82(1):1–16.

Yang, L., Wang, H., Liu, L., and Xie, A. (2018). The Role of Insulin/IGF-1/PI3K/Akt/GSK3B Signaling in Parkinson's Disease Dementia. *Frontiers in Neuroscience*, 12:73.
