# Supplementary material for: Sensitivity analysis of a mathematical model of Alzheimer's disease progression unveils important causal pathways
Source: Front Neuroinform. 2025 Jul 23;19:1590968. doi: 10.3389/fninf.2025.1590968 (PMC12325246; doi:10.3389/fninf.2025.1590968)
Supplement: Supplementary file 2 [file Supplementary_file_2.pdf]

A Appendix: Sources of Model Parameters Determined from Scientific Literature.

This appendix lists all the parameters used in the equations for amyloid-beta, NFTs, dead neurons, microglia, macrophages, cytokines, and chemokines, sourced from the literature.

Parameter	Signification	Source
ρ_{cerveau}	Brain density (g/mL)	(National Institute of Standards and Technology, 2017)
N_0 (woman)	Neuron density in women (g/mL)	(Pelvig et al., 2008)
N_0 (man)	Neuron density in men (g/mL)	(Pelvig et al., 2008)
A_0 (woman)	Astrocyte density in women (g/mL)	(Pelvig et al., 2008)
A_0 (man)	Astrocyte density in men (g/mL)	(Pelvig et al., 2008)
	**Amyloid-beta (Aβ)**	
M_{ABm}	Molar mass of Aβ monomer	(Raskatov, 2019)
λ_{AB^i}	Creation rate of Aβ inside neurons	(Hao and Friedman, 2016; Lindstrom et al., 2021)
δ_{AP^i}	Effect of APOE4 on Aβ creation inside neurons	(Roher et al., 2009)
d_{AB^i}	Degradation rate of Aβ inside neurons	(Saido and Leissring, 2012; Cirrito et al., 2003; Savage et al., 1998)
$\lambda_{\text{AB}^o_m}$	Aβ monomer creation outside neuron	(Hao and Friedman, 2016; Lindstrom et al., 2021)
δ_{APm}	Quantifies the impact of the presence of the APOE4 gene on the creation of extracellular Aβ by APP	(Roher et al., 2009)
$\lambda_{AAB^o_m}$	Aβ monomer creation by astrocytes	(Zhao et al., 2011; Blasko et al., 2000)
$\kappa_{\text{AB}^o_m\text{AB}^o_o}$	Monomer to oligomer conversion rate	(Garai and Frieden, 2013)
$\delta_{\text{AP}_{mo}}$	APOE4 effect on monomer–oligomer conversion	(Hashimoto et al., 2012)
$\kappa_{\text{ABoo}\to\text{ABpo}}$	Oligomer to plaque conversion rate	(Garai et al., 2014; Raskatov, 2019)
$d_{\text{AB}^o_o}$	Oligomer degradation rate	(Garai and Frieden, 2013)
$d_{\text{AB}^o_m}$	Degradation of $d_{\text{AB}^o_m}$	(Garai and Frieden, 2013)
$d_{\hat{M}_{\text{anti}}\text{AB}^o_p}$	Plaque degradation by anti-inflammatory macrophages	(Majumdar et al., 2008)
$d_{M_{\text{anti}}\text{AB}^o_p}$	Plaque degradation by anti-inflammatory microglia	(DeWitt et al., 1998)
$\delta_{\text{AP}_{dp}}$	APOE4 effect on plaque degradation	(Zhao et al., 2009)
$K_{\text{AB}^o_p}$	Michaelis-Menten constant for plaque degradation	(Roberts et al., 2017)
	**GSK-3, tau protein and NFTs**	
Ins_0	Normal insulin concentration in brain at 30 years old	(Chamberland et al., 2024)
$Ins(t)$	Insulin concentration is a function of age	(Gray et al., 2014; Wallum et al., 1987; Blázquez et al., 2014; Poggiogalle et al., 2018)
d_G	Degradation rate OF GSK-3	(Domínguez et al., 2012)
G_0 (men)	Normal GSK-3 concentration, based on brain density	(Knight et al., 2021)
G_0 (women)	Normal GSK-3 concentration, based on brain density	(Pandey and DeGrado, 2016)
λ_{InsG} (men)	Creation rate of GSK-3 for men induced by insulin	(Knight et al., 2021; Domínguez et al., 2012)
λ_{InsG} (women)	Creation rate of GSK-3 for women induced by insulin	(Pandey and DeGrado, 2016; Domínguez et al., 2012)
λ_τ	Baseline tau phosphorylation rate	(Sato et al., 2018)
$\lambda_{G\tau}$	Tau phosphorylation by GSK-3	(Sjögren et al., 2001; Park and Bowers, 2010)
$\kappa_{\tau Fi}$	Conversion rate of tau to intracellular NFTs	(Townsend et al., 2020; Alavi Naini and Soussi-Yanicostas, 2018; Goedert and Jakes, 1990)
d_τ	Tau degradation rate, based on half-life	(Sato et al., 2018)

Parameter	Signification	Source
d_{Fi}	Intracellular NFT degradation	(Kompoliti and Verhagen, 2010; Hao and Friedman, 2016)
κ_{MFo}	Max rate of extracellular NFT degradation by microglia	(Luo et al., 2015)
K_{Manti} (men)	Half-max concentration of M_{anti} for NFT degradation in men	(Pelvig et al., 2008)
K_{Manti} (women)	Half-max concentration of M_{anti} for NFT degradation in women	(Pelvig et al., 2008)
d_{Fo}	Extracellular NFT degradation rate	(Pelvig et al., 2008)
	**Neurons**	
d_{FiN}	Max neuron death rate from F_i	(Schwab et al., 1999)
K_{Fi}	Half-max concentration of F_i for neuron death	(Khatoon et al., 1992)
d_{TaN}	Max neuron death rate from TNF-α	(Potvin et al., 2021)
K_{Ta}	Half-max concentration of TNF-α for neuron death	(Csuka et al., 1999; Zhao et al., 2003)
K_{I10}	Half-max IL-10 concentration inhibiting TNF-α-induced death	(Hayakata et al., 2004; Kirchhoff et al., 2008)
	**Astrocytes**	
A_{max}	Max astrocyte density, assumed equal to initial	(Pelvig et al., 2008)
$\kappa_{T_\alpha A}$	Astrocyte activation rate by TNF-α	(Liddelow and Barres, 2017; Russ et al., 2021)
$\kappa_{AB^o_p A}$	Astrocyte activation by Aβ plaque	(Csuka et al., 1999; Zhao et al., 2011)
d_A	Astrocyte death rate	(Sofroniew, 2020)
	**Microglia**	
$d_{M\,pro}$	Deactivation rate of pro-inflammatory microglia	(Réu et al., 2017)
$d_{M\,anti}$	Deactivation rate of anti-inflammatory microglia	(Hao and Friedman, 2016)
$\kappa_{F_o M}$	Maximal rate of microglia activation by extracellular NFTs	(Hao and Friedman, 2016)
$\kappa_{AB^o_o M}$	Maximal rate of microglia activation by Aβ oligomers	(Hao and Friedman, 2016)
K_{F_o}	Concentration of extracellular NFTs at which microglia activation is half maximal	(Roberts et al., 2017)
$\kappa_{T_B M_{pro}}$	Concentration of Aβ oligomers extracellular at which microglia activation is half maximal	(Amato and Arnold, 2021)
$K_{T_B M}$	Half saturation constant of TGF-β for $\mathrm{M_{anti}}$ to $\mathrm{M_{pro}}$ conversion	(Tarkowski et al., 2001; Swardfager et al., 2010; Li et al., 2007)
$\kappa_{T_\alpha M_{\mathrm{anti}}}$	Max rate of conversion $\mathrm{M_{anti}}$ to $\mathrm{M_{pro}}$ under signaling of TNF-α	(Tang and Le, 2016; Amato and Arnold, 2021)
$K_{T_\alpha M}$	Half-saturation constant of TNF-α for $\mathrm{M_{anti}}$ to $\mathrm{M_{pro}}$ conversion	(Csuka et al., 1999)
	**Macrophages**	
$\kappa_{P\hat{M}}$	Maximum rate of macrophage import into the brain under MCP-1 signaling	(Semple et al., 2010; Hughes et al., 2002; Fumagalli et al., 2015; Deshmane et al., 2009)
K_P	MCP-1 concentration at which the macrophage import rate into the brain is half-maximal	(Jeppsson et al., 2019; Johansson et al., 2017; Rosén et al., 2014; Westin et al., 2012)
$\hat{M}_{\mathrm{max}}$	Total possible concentration of activated macrophages	(Borda et al., 2008; Serrano-Pozo et al., 2013)
$\kappa_{T_\beta \hat{M}_{\mathrm{pro}}}$	Maximum rate of conversion from pro-inflammatory to anti-inflammatory macrophages ($\hat{M}_{\mathrm{pro}} \to \hat{M}_{\mathrm{anti}}$) under TGF-$\beta$ signaling	(Orihuela et al., 2016; Wang et al., 2021; Song et al., 2022)

Parameter	Signification	Source
$\kappa_{T_\alpha \hat{M}_{\text{anti}}}$	Maximum rate of conversion from anti-inflammatory to pro-inflammatory macrophages ($\hat{M}_{\text{anti}} \to \hat{M}_{\text{pro}}$) under TNF-$\alpha$ signaling	(Tang and Le, 2016; Khallou-Laschet et al., 2010)
$d_{\hat{M}_{\text{pro}}}$	Death rate of pro-inflammatory macrophages	(Réu et al., 2017)
$d_{\hat{M}_{\text{anti}}}$	Death rate of anti-inflammatory macrophages	(Réu et al., 2017)
**Cytokines and Chimiokines**		
$\kappa_{M_{\text{anti}} T_\beta}$	TGF-β production rate by anti-inflammatory microglia	(Cao et al., 2010; Fadok et al., 1998)
$\kappa_{\hat{M}_{\text{anti}} T_\beta}$	TGF-β production rate by anti-inflammatory macrophages	(Cao et al., 2010; Fadok et al., 1998)
d_{T_β}	TGF-β degradation rate	(Wakefield et al., 1990)
$\kappa_{M_{\text{anti}} I10}$	IL-10 production rate by anti-inflammatory microglia	(Mia et al., 2014; Hao and Friedman, 2016)
$\kappa_{\hat{M}_{\text{anti}} I10}$	IL-10 production rate by anti-inflammatory macrophages	(Mia et al., 2014; Hao and Friedman, 2016)
d_{I10}	IL-10 degradation rate	(Huhn et al., 1997)
$\kappa_{M_{\text{pro}} T_\alpha}$	TNF-α production rate by pro-inflammatory microglia	(Liddelow et al., 2017; Wang and Wyss-Coray, 2015; Fadok et al., 1998)
$\kappa_{\hat{M}_{\text{pro}} T_\alpha}$	TNF-α production rate of pro-inflammatory macrophages	(Liddelow et al., 2017; Wang and Wyss-Coray, 2015; Fadok et al., 1998)
d_{T_α}	TNF-α degradation rate	(Zahn and Greischel, 1989; Oliver et al., 1993)
$\kappa_{\hat{M}_{\text{pro}} P}$	production rate by MCP-1 by active astrocytes	(Orihuela et al., 2016; Bardi et al., 2018; Lee et al., 2018; Yoshimura et al., 2016)
$\kappa_{M_{\text{pro}} P}$	production rate of MCP-1 by pro-inflammatory microglia	(Orihuela et al., 2016; Bardi et al., 2018; Lee et al., 2018; Yoshimura et al., 2016)
κ_{AP}	Production rate of MCP-1 by active astrocytes	(Lee et al., 2018)
d_P	MCP-1 degradation rate	(Zhu et al., 2005)

References

Alavi Naini, S. M. and Soussi-Yanicostas, N. (2018). Heparan sulfate as a therapeutic target in tauopathies: insights from zebrafish. *Frontiers in cell and developmental biology*, 6:163.

Amato, S. and Arnold, A. (2021). Modeling microglia activation and inflammation-based neuroprotectant strategies during ischemic stroke. *Bulletin of Mathematical Biology*, 83(6):72.

Bardi, G. T., Smith, M. A., and Hood, J. L. (2018). Melanoma exosomes promote mixed m1 and m2 macrophage polarization. *Cytokine*, 105:63–72.

Blasko, I., Veerhuis, R., Stampfer-Kountchev, M., Saurwein-Teissl, M., Eikelenboom, P., and Grubeck-Loebenstein, B. (2000). Costimulatory effects of interferon-γ and interleukin-1β or tumor necrosis factor α on the synthesis of aβ1-40 and aβ1-42 by human astrocytes. *Neurobiology of disease*, 7(6):682–689.

Blázquez, E., Velázquez, E., Hurtado-Carneiro, V., and Ruiz-Albusac, J. M. (2014). Insulin in the brain: its pathophysiological implications for states related with central insulin resistance, type 2 diabetes and alzheimer's disease. *Frontiers in endocrinology*, 5:161.

Borda, J. T., Alvarez, X., Mohan, M., Hasegawa, A., Bernardino, A., Jean, S., Aye, P., and Lackner, A. A. (2008). Cd163, a marker of perivascular macrophages, is up-regulated by microglia in simian immunodeficiency virus encephalitis after haptoglobin-hemoglobin complex stimulation and is suggestive of breakdown of the blood-brain barrier. *The American journal of pathology*, 172(3):725–737.

Cao, Q., Wang, Y., Zheng, D., Sun, Y., Wang, Y., Lee, V. W., Zheng, G., Tan, T. K., Ince, J., Alexander, S. I., et al. (2010). Il-10/tgf-β–modified macrophages induce regulatory t cells and protect against adriamycin nephrosis. *Journal of the American Society of Nephrology*, 21(6):933–942.

Chamberland, É., Moravveji, S., Doyon, N., and Duchesne, S. (2024). A computational model of alzheimer's disease at the nano, micro, and macroscales. *Frontiers in Neuroinformatics*, 18:1348113.

Cirrito, J. R., May, P. C., O'Dell, M. A., Taylor, J. W., Parsadanian, M., Cramer, J. W., Audia, J. E., Nissen, J. S., Bales, K. R., Paul, S. M., et al. (2003). In vivo assessment of brain interstitial fluid with microdialysis reveals plaque-associated changes in amyloid-β metabolism and half-life. *Journal of Neuroscience*, 23(26):8844–8853.

Csuka, E., Morganti-Kossmann, M. C., Lenzlinger, P. M., Joller, H., Trentz, O., and Kossmann, T. (1999). Il-10 levels in cerebrospinal fluid and serum of patients with severe traumatic brain injury: relationship to il-6, tnf-α, tgf-β1 and blood–brain barrier function. *Journal of neuroimmunology*, 101(2):211–221.

Deshmane, S. L., Kremlev, S., Amini, S., and Sawaya, B. E. (2009). Monocyte chemoattractant protein-1 (mcp-1): an overview. *Journal of Interferon & Cytokine Research*, 29(6):313–326.

DeWitt, D. A., Perry, G., Cohen, M., Doller, C., and Silver, J. (1998). Astrocytes regulate microglial phagocytosis of senile plaque cores of alzheimer's disease. *Experimental neurology*, 149(2):329–340.

Domínguez, J. M., Fuertes, A., Orozco, L., del Monte-Millán, M., Delgado, E., and Medina, M. (2012). Evidence for irreversible inhibition of glycogen synthase kinase-3β by tideglusib. *Journal of Biological Chemistry*, 287(2):893–904.

Fadok, V. A., Bratton, D. L., Konowal, A., Freed, P. W., Westcott, J. Y., Henson, P. M., et al. (1998). Macrophages that have ingested apoptotic cells in vitro inhibit proinflammatory cytokine production through autocrine/paracrine mechanisms involving tgf-beta, pge2, and paf. *The Journal of clinical investigation*, 101(4):890–898.

Fumagalli, S., Perego, C., Pischiutta, F., Zanier, E. R., and De Simoni, M.-G. (2015). The ischemic environment drives microglia and macrophage function. *Frontiers in neurology*, 6:81.

Garai, K. and Frieden, C. (2013). Quantitative analysis of the time course of aβ oligomerization and subsequent growth steps using tetramethylrhodamine-labeled aβ. *Proceedings of the National Academy of Sciences*, 110(9):3321–3326.

Garai, K., Verghese, P. B., Baban, B., Holtzman, D. M., and Frieden, C. (2014). The binding of apolipoprotein e to oligomers and fibrils of amyloid-β alters the kinetics of amyloid aggregation. *Biochemistry*, 53(40):6323–6331.

Goedert, M. and Jakes, R. (1990). Expression of separate isoforms of human tau protein: correlation with the tau pattern in brain and effects on tubulin polymerization. *The EMBO journal*, 9(13):4225.

Gray, S. M., Meijer, R. I., and Barrett, E. J. (2014). Insulin regulates brain function, but how does it get there? *Diabetes*, 63(12):3992–3997.

Hao, W. and Friedman, A. (2016). Mathematical model on alzheimer's disease. *BMC Systems Biology*, 10(1):108.

Hashimoto, T., Serrano-Pozo, A., Hori, Y., Adams, K. W., Takeda, S., Banerji, A. O., Mitani, A., Joyner, D., Thyssen, D. H., Bacskai, B. J., et al. (2012). Apolipoprotein e, especially apolipoprotein e4, increases the oligomerization of amyloid β peptide. *Journal of Neuroscience*, 32(43):15181–15192.

Hayakata, T., Shiozaki, T., Tasaki, O., Ikegawa, H., Inoue, Y., Toshiyuki, F., Hosotubo, H., Kieko, F., Yamashita, T., Tanaka, H., et al. (2004). Changes in csf s100b and cytokine concentrations in early-phase severe traumatic brain injury. *Shock*, 22(2):102–107.

Hughes, P. M., Allegrini, P. R., Rudin, M., Perry, V. H., Mir, A. K., and Wiessner, C. (2002). Monocyte chemoattractant protein-1 deficiency is protective in a murine stroke model. *Journal of Cerebral Blood Flow & Metabolism*, 22(3):308–317.

Huhn, R. D., Radwanski, E., Gallo, J., Affrime, M. B., Sabo, R., Gonyo, G., Monge, A., and Cutler, D. L. (1997). Pharmacodynamics of subcutaneous recombinant human interleukin-10 in healthy volunteers. *Clinical Pharmacology & Therapeutics*, 62(2):171–180.

Jeppsson, A., Wikkelsö, C., Blennow, K., Zetterberg, H., Constantinescu, R., Remes, A. M., Herukka, S.-K., Rauramaa, T., Nagga, K., Leinonen, V., et al. (2019). Csf biomarkers distinguish idiopathic normal pressure hydrocephalus from its mimics. *Journal of Neurology, Neurosurgery & Psychiatry*, 90(10):1117–1123.

Johansson, P., Almqvist, E. G., Bjerke, M., Wallin, A., Johansson, J.-O., Andreasson, U., Blennow, K., Zetterberg, H., and Svensson, J. (2017). Reduced cerebrospinal fluid concentration of apolipoprotein ai in patients with alzheimer's disease. *Journal of Alzheimer's disease*, 59(3):1017–1026.

Khallou-Laschet, J., Varthaman, A., Fornasa, G., Compain, C., Gaston, A.-T., Clement, M., Dussiot, M., Levillain, O., Graff-Dubois, S., Nicoletti, A., et al. (2010). Macrophage plasticity in experimental atherosclerosis. *PloS one*, 5(1):e8852.

Khatoon, S., Grundke-Iqbal, I., and Iqbal, K. (1992). Brain levels of microtubule-associated protein τ are elevated in alzheimer's disease: A radioimmuno-slot-blot assay for nanograms of the protein. *Journal of neurochemistry*, 59(2):750–753.

Kirchhoff, C., Buhmann, S., Bogner, V., Stegmaier, J., Leidel, B., Braunstein, V., Mutschler, W., and Biberthaler, P. (2008). Cerebrospinal il-10 concentration is elevated in non-survivors as compared to survivors after severe traumatic brain injury. *Eur J Med Res*, 13(10):464–468.

Knight, A. C., Varlow, C., Tong, J., and Vasdev, N. (2021). In vitro and in vivo evaluation of gsk-3 radioligands in alzheimer's disease: preliminary evidence of sex differences. *ACS Pharmacology & Translational Science*, 4(4):1287–1294.

Kompoliti, K. and Verhagen, L. (2010). *Encyclopedia of movement disorders*, volume 1. Academic Press.

Lee, S., Lee, J., Kim, J., Lee, H., Kim, Y., Kang, S., Kim, S., Kim, S., Kim, S., Kim, S., et al. (2018). Plasma mcp-1 levels correlate with neuroinflammation and the severity of alzheimer's disease. *Journal of Alzheimer's Disease*, 63(1):1–10.

Li, X., Miyajima, M., Jiang, C., and Arai, H. (2007). Expression of tgf-βs and tgf-β type ii receptor in cerebrospinal fluid of patients with idiopathic normal pressure hydrocephalus. *Neuroscience letters*, 413(2):141–144.

Liddelow, S. A. and Barres, B. A. (2017). Reactive astrocytes: production, function, and therapeutic potential. *Immunity*, 46(6):957–967.

Liddelow, S. A., Guttenplan, K. A., Clarke, L. E., Bennett, F. C., Bohlen, C. J., Schirmer, L., Bennett, M. L., Münch, A. E., Chung, W.-S., Peterson, T. C., et al. (2017). Neurotoxic reactive astrocytes are induced by activated microglia. *Nature*, 541(7638):481–487.

Lindstrom, M. R., Chavez, M. B., Gross-Sable, E. A., Hayden, E. Y., and Teplow, D. B. (2021). From reaction kinetics to dementia: A simple dimer model of alzheimer's disease etiology. *PLOS Computational Biology*, 17(5):e1009114.

Luo, W., Liu, W., Hu, X., Hanna, M., Caravaca, A., and Paul, S. M. (2015). Microglial internalization and degradation of pathological tau is enhanced by an anti-tau monoclonal antibody. *Scientific reports*, 5(1):11161.

Majumdar, A., Chung, H., Dolios, G., Wang, R., Asamoah, N., Lobel, P., and Maxfield, F. R. (2008). Degradation of fibrillar forms of alzheimer's amyloid β-peptide by macrophages. *Neurobiology of aging*, 29(5):707–715.

Mia, S., Warnecke, A., Zhang, X.-M., Malmström, V., and Harris, R. A. (2014). An optimized protocol for human m2 macrophages using m-csf and il-4/il-10/tgf-β yields a dominant immunosuppressive phenotype. *Scandinavian journal of immunology*, 79(5):305–314.

National Institute of Standards and Technology (2017). Composition of brain (icrp). https://physics.nist.gov/cgi-bin/Star/compos.pl?matno=123. Accessed: 2022-05-17.

Oliver, J. C., Bland, L., Oettinger, C. W., Arduino, M. J., McAllister, S., Aguero, S., and Favero, M. (1993). Cytokine kinetics in an in vitro whole blood model following an endotoxin challenge. *Lymphokine and cytokine research*, 12(2).

Orihuela, R., McPherson, C. A., and Harry, G. J. (2016). Microglial m1/m2 polarization and metabolic states. *British journal of pharmacology*, 173(4):649–665.

Pandey, M. K. and DeGrado, T. R. (2016). Glycogen synthase kinase-3 (gsk-3)-targeted therapy and imaging. *Theranostics*, 6(4):571.

Park, K. M. and Bowers, W. J. (2010). Tumor necrosis factor-alpha mediated signaling in neuronal homeostasis and dysfunction. *Cellular signalling*, 22(7):977–983.

Pelvig, D. P., Pakkenberg, H., Stark, A. K., and Pakkenberg, B. (2008). Neocortical glial cell numbers in human brains. *Neurobiology of aging*, 29(11):1754–1762.

Poggiogalle, E., Jamshed, H., and Peterson, C. M. (2018). Circadian regulation of glucose, lipid, and energy metabolism in humans. *Metabolism*, 84:11–27.

Potvin, O., Dieumegarde, L., Duchesne, S., Initiative, A. D. N., CIMA-Q, and groups, C. (2021). Nomis: Quantifying morphometric deviations from normality over the lifetime of the adult human brain. *BioRxiv*, pages 2021–01.

Raskatov, J. A. (2019). What is the “relevant” amyloid β42 concentration? *ChemBioChem*, 20(13):1725–1726.

Réu, P., Khosravi, A., Bernard, S., Mold, J. E., Salehpour, M., Alkass, K., Perl, S., Tisdale, J., Possnert, G., Druid, H., et al. (2017). The lifespan and turnover of microglia in the human brain. *Cell reports*, 20(4):779–784.

Roberts, B. R., Lind, M., Wagen, A. Z., Rembach, A., Frugier, T., Li, Q.-X., Ryan, T. M., McLean, C. A., Doecke, J. D., Rowe, C. C., et al. (2017). Biochemically-defined pools of amyloid-β in sporadic alzheimer’s disease: correlation with amyloid pet. *Brain*, 140(5):1486–1498.

Roher, A. E., Esh, C. L., Kokjohn, T. A., Castaño, E. M., Van Vickle, G. D., Kalback, W. M., Patton, R. L., Luehrs, D. C., Daugs, I. D., Kuo, Y.-M., et al. (2009). Amyloid beta peptides in human plasma and tissues and their significance for alzheimer’s disease. *Alzheimer’s & Dementia*, 5(1):18–29.

Rosén, C., Andersson, C.-H., Andreasson, U., Molinuevo, J. L., Bjerke, M., Rami, L., Lladó, A., Blennow, K., and Zetterberg, H. (2014). Increased levels of chitotriosidase and ykl-40 in cerebrospinal fluid from patients with alzheimer’s disease. *Dementia and geriatric cognitive disorders extra*, 4(2):297–304.

Russ, K., Teku, G., Bousset, L., Redeker, V., Piel, S., Savchenko, E., Pomeshchik, Y., Savistchenko, J., Stummann, T. C., Azevedo, C., et al. (2021). Tnf-α and α-synuclein fibrils differently regulate human astrocyte immune reactivity and impair mitochondrial respiration. *Cell reports*, 34(12).

Saido, T. and Leissring, M. A. (2012). Proteolytic degradation of amyloid β-protein. *Cold Spring Harbor perspectives in medicine*, 2(6):a006379.

Sato, C., Barthélemy, N. R., Mawuenyega, K. G., Patterson, B. W., Gordon, B. A., Jockel-Balsarotti, J., Sullivan, M., Crisp, M. J., Kasten, T., Kirmess, K. M., et al. (2018). Tau kinetics in neurons and the human central nervous system. *Neuron*, 97(6):1284–1298.

Savage, M. J., Trusko, S. P., Howland, D. S., Pinsker, L. R., Mistretta, S., Reaume, A. G., Greenberg, B. D., Siman, R., and Scott, R. W. (1998). Turnover of amyloid β-protein in mouse brain and acute reduction of its level by phorbol ester. *Journal of Neuroscience*, 18(5):1743–1752.

Schwab, C., Schulzer, M., Steele, J. C., and McGeer, P. L. (1999). On the survival time of a tangled neuron in the hippocampal ca4 region in parkinsonism dementia complex of guam. *Neurobiology of aging*, 20(1):57–63.

Semple, B. D., Bye, N., Rancan, M., Ziebell, J. M., and Morganti-Kossmann, M. C. (2010). Role of ccl2 (mcp-1) in traumatic brain injury (tbi): evidence from severe tbi patients and ccl2-/- mice. *Journal of Cerebral Blood Flow & Metabolism*, 30(4):769–782.

Serrano-Pozo, A., Gómez-Isla, T., Growdon, J. H., Frosch, M. P., and Hyman, B. T. (2013). A phenotypic change but not proliferation underlies glial responses in alzheimer disease. *The American journal of pathology*, 182(6):2332–2344.

Sjögren, M., Davidsson, P., Tullberg, M., Minthon, L., Wallin, A., Wikkelso, C., Granérus, A.-K., Vanderstichele, H., Vanmechelen, E., and Blennow, K. (2001). Both total and phosphorylated tau are increased in alzheimer’s disease. *Journal of Neurology, Neurosurgery & Psychiatry*, 70(5):624–630.

Sofroniew, M. V. (2020). Astrocyte reactivity: subtypes, states, and functions in cns innate immunity. *Trends in immunology*, 41(9):758–770.

Song, W., Koo, E., and Koh, S. (2022). Platelet-derived tgf-b1 promotes microglial polarization and inhibits ab clearance by upregulating smad2/3/4 in alzheimer’s disease. *Journal of Neuroinflammation*, 19(1):1–14.

Swardfager, W., Lanctôt, K., Rothenburg, L., Wong, A., Cappell, J., and Herrmann, N. (2010). A meta-analysis of cytokines in alzheimer’s disease. *Biological psychiatry*, 68(10):930–941.

Tang, Y. and Le, W. (2016). Differential roles of m1 and m2 microglia in neurodegenerative diseases. *Molecular Neurobiology*, 53(2):1181–1194.

Tarkowski, E., Wallin, A., Regland, B., Blennow, K., and Tarkowski, A. (2001). Local and systemic gm-csf increase in alzheimer’s disease and vascular dementia. *Acta neurologica scandinavica*, 103(3):166–174.

Townsend, D., Fullwood, N. J., Yates, E. A., and Middleton, D. A. (2020). Aggregation kinetics and filament structure of a tau fragment are influenced by the sulfation pattern of the cofactor heparin. *Biochemistry*, 59(41):4003–4014.

Wakefield, L. M., Winokur, T. S., Hollands, R. S., Christopherson, K., Levinson, A. D., Sporn, M. B., et al. (1990). Recombinant latent transforming growth factor beta 1 has a longer plasma half-life in rats than active transforming growth factor beta 1, and a different tissue distribution. *The Journal of clinical investigation*, 86(6):1976–1984.

Wallum, B., Taborsky Jr, G., Porte Jr, D., Figlewicz, D., Jacobson, L., Beard, J., Ward, W., and Dorsa, D. (1987). Cerebrospinal fluid insulin levels increase during intravenous insulin infusions in man. *The Journal of Clinical Endocrinology & Metabolism*, 64(1):190–194.

Wang, W. and Wyss-Coray, T. (2015). The role of anti-inflammatory cytokines in neurodegenerative diseases. *Nature Reviews Neurology*, 11(9):528–539.

Wang, Y., Cella, M., Mallinson, K., Ulrich, J., Young, K., Robinette, M., Gilfillan, S., Krishnan, G., Sudhakar, S., Zinselmeyer, B., et al. (2021). Microglia and alzheimer's disease: A review of the literature. *Journal of Alzheimer's Disease*, 82(1):1–16.

Westin, K., Buchhave, P., Nielsen, H., Minthon, L., Janciauskiene, S., and Hansson, O. (2012). Ccl2 is associated with a faster rate of cognitive decline during early stages of alzheimer's disease. *PloS one*, 7(1):e30525.

Yoshimura, T., Imamichi, T., Weiss, J. M., Sato, M., Li, L., Matsukawa, A., and Wang, J. M. (2016). Induction of monocyte chemoattractant proteins in macrophages via the production of granulocyte/macrophage colony-stimulating factor by breast cancer cells. *Frontiers in immunology*, 7:2.

Zahn, G. and Greischel, A. (1989). Pharmacokinetics of tumor necrosis factor alpha after intravenous administration in rats. dose dependence and influence of tumor necrosis factor beta. *Arzneimittel-forschung*, 39(9):1180–1182.

Zhao, J., O'Connor, T., and Vassar, R. (2011). The contribution of activated astrocytes to aβ production: implications for alzheimer's disease pathogenesis. *Journal of neuroinflammation*, 8:1–17.

Zhao, L., Lin, S., Bales, K. R., Gelfanova, V., Koger, D., DeLong, C., Hale, J., Liu, F., Hunter, J. M., and Paul, S. M. (2009). Macrophage-mediated degradation of β-amyloid via an apolipoprotein e isoform-dependent mechanism. *Journal of Neuroscience*, 29(11):3603–3612.

Zhao, M., Cribbs, D. H., Anderson, A. J., Cummings, B. J., Su, J. H., Wasserman, A. J., and Cotman, C. W. (2003). The induction of the tnfα death domain signaling pathway in alzheimer's disease brain. *Neurochemical research*, 28:307–318.

Zhu, C., Ying, D., Zhou, D., Mi, J., Zhang, W., Chang, Q., and Li, L. (2005). Expression of tgf-beta1 in smooth muscle cells regulates endothelial progenitor cells migration and differentiation1. *Journal of Surgical Research*, 125(2):151–156.
